# Increased Endoplasmic Reticulum Stress and Decreased Proteasomal Function in Lafora Disease Models Lacking the Phosphatase Laforin

**DOI:** 10.1371/journal.pone.0005907

**Published:** 2009-06-16

**Authors:** Santiago Vernia, Teresa Rubio, Miguel Heredia, Santiago Rodríguez de Córdoba, Pascual Sanz

**Affiliations:** 1 Instituto de Biomedicina de Valencia, CSIC and CIBER de Enfermedades Raras (CIBERER), Valencia, Spain; 2 Centro de Investigaciones Biológicas, CSIC and CIBER de Enfermedades Raras (CIBERER), Madrid, Spain; National Institutes of Health, United States of America

## Abstract

**Background:**

Lafora progressive myoclonus epilepsy (Lafora disease; LD) is a fatal autosomal recessive neurodegenerative disorder caused by loss-of-function mutations in either the *EPM2A* gene, encoding the dual specificity phosphatase laforin, or the *EPM2B* gene, encoding the E3-ubiquitin ligase malin. Previously, we and others have shown that both proteins form a functional complex that regulates glycogen synthesis by a novel mechanism involving ubiquitination and proteasomal degradation of at least two proteins, glycogen synthase and R5/PTG. Since laforin and malin localized at the endoplasmic reticulum (ER) and their regulatory role likely extend to other proteins unrelated to glycogen metabolism, we postulated that their absence may also affect the ER-unfolded protein response pathway.

**Methodology/Principal Findings:**

Here, we demonstrate that siRNA silencing of laforin in Hek293 and SH-SY5Y cells increases their sensitivity to agents triggering ER-stress, which correlates with impairment of the ubiquitin-proteasomal pathway and increased apoptosis. Consistent with these findings, analysis of tissue samples from a LD patient lacking laforin, and from a laforin knockout (Epm2a-/-) mouse model of LD, demonstrates constitutive high expression levels of ER-stress markers BIP/Grp78, CHOP and PDI, among others.

**Conclusions/Significance:**

We demonstrate that, in addition to regulating glycogen synthesis, laforin and malin play a role protecting cells from ER-stress, likely contributing to the elimination of unfolded proteins. These data suggest that proteasomal dysfunction and ER-stress play an important role in the pathogenesis of LD, which may offer novel therapeutic approaches for this fatal neurodegenerative disorder.

## Introduction

Lafora progressive myoclonus epilepsy (LD, OMIM 254780) is a fatal autosomal recessive neurodegenerative disorder characterized by the presence of glycogen-like intracellular inclusions named Lafora bodies (see [1] and [2] for review). LD initially manifests during adolescence with generalized tonic-clonic seizures, myoclonus, absences, drop attacks and visual hallucinations. As the disease proceeds, a rapidly progressive dementia with apraxia, aphasia and visual loss ensues, leading patients to a vegetative state and death, usually within the first decade from onset of the first symptoms ([1] and [2]). Mutations causing LD have been identified in two genes, *EPM2A* ([3], [4]) and *EPM2B (NHLRC1)* [5], although there is evidence for a third locus [6]. *EPM2A* encodes laforin, a dual specificity phosphatase of 331 amino acids with a functional carbohydrate binding domain at the N-terminus ([7], [8]). *EPM2B* encodes malin, an E3-ubiquitin ligase of 395 amino acids with a RING finger domain at the N-terminus and six NHL domains in the C-terminal region which are involved in protein-protein interactions ([5], [9], [10]). We and others have recently described that laforin interacts physically with malin and that laforin recruits specific substrates to be ubiquitinated by malin, targeting them for proteasomal degradation ([9], [10], [11]). In fact, it has been described that the laforin-malin complex is involved in the degradation of the muscle isoform of glycogen synthase [12], the glycogen debranching enzyme (AGL) [13], and some glycogen targeting subunits of type 1 protein phosphatase (PP1), such as R5/PTG ([11], [12], [14]) and R6 [14]. Recently, an alternative function of laforin on glycogen homeostasis has been described ([15], [16]). In this case, laforin acts as a phosphatase of complex carbohydrates and it has been proposed that this function might be necessary for the maintenance of normal cellular glycogen ([17], [18]). Taken together, these results define the importance of the laforin-malin complex in regulating glycogen biosynthesis. This is consistent with the accumulation of glycogen-like intracellular inclusions (Lafora bodies), as one of the histological determinants of LD. However, it is still under debate whether the accumulation of Lafora bodies is the cause of the disease or if they are only the result of a previously established neurodegeneration.

Lafora bodies contain around 90% glucose polymers and 6% protein ([19], [20]). They stain positive for anti-ubiquitin and anti-advanced glycation end products antibodies [21], which suggest that they contain misfolded proteins destined for degradation ([9], [21]). For this reason, it has been proposed that LD is a disorder of protein clearance [2]. Consistent with this idea, it has been described recently that laforin and malin form centrosomal aggregates when the cells are treated with proteasomal inhibitors, being these aggregates immunoreactive to ubiquitin, ubiquitin-conjugating enzymes, proteasomal subunits and chaperones, demonstrating their aggresome-like properties [22]. It is known that aggresome formation is a general cell response which occurs when the capacity of the proteasome is exceeded by the production of misfolded proteins. These proteins have a strong tendency to aggregate, which results in an impairment of proteasomal function since the capacity of the proteolytic machinery is saturated by non-degradable material [23].

Impairment of proteasomal function leads, among other effects, to the induction of stress at the endoplasmic reticulum (ER-stress), probably by the inhibition of ER-associated degradation (ERAD, see below), which eventually leads to apoptotic cell death [24]. ER function is highly sensitive to stresses that perturb cellular energy levels, red-ox status or Ca++ concentration. Such stresses reduce the folding capacity of the ER, which results in the accumulation and aggregation of unfolded proteins in the lumen. These events trigger a signal responsible for the activation of the unfolded protein response (UPR), resulting in restoration of ER function. In addition, unfolded proteins are retrotranslocated to the cytosol, polyubiquitinated and degraded by the proteasome by the ER-associated degradation (ERAD) pathway. The UPR pathway is characterized by the activation of two ER-resident kinases (PKR-like ER kinase, PERK; and the inositol-requiring protein 1, IRE1) and the translocation to the Golgi apparatus and subsequent cleavage of ATF6, a transmembrane ER-resident protein with a cytosolic domain with transcriptional activity. These components induce signaling cascades that lead to the overexpression of characteristic UPR-mediators, such as the heat shock protein BIP/Grp78, the protein disulphide isomerase PDI, the transcriptional factor CHOP (a member of the C/EBP family of bZIP transcription factors that induce apoptosis) and the phosphorylated form of the eukaryotic initiation factor 2 alpha (p-EIF2α), among others (see [25], [26], [27], [28], for review).

Since laforin and malin are localized at the ER ([22], [29], [30]), we tested whether they were affecting the ER-unfolded protein response pathway. In this work, we show that under conditions of laforin depletion, two different human cell lines Hek293 and SH-SY5Y, increase their sensitivity to agents triggering ER-stress, and this correlates with impairment of the ubiquitin-proteasomal pathway and increased apoptosis. Additionally, in both a mouse Epm2a-/- model and in necropsies from a human LD patient with mutations in the *EPM2A* gene, we detected higher levels of ER-stress markers, indicating that in the absence of laforin, cells were under permanent conditions of ER-stress, probably due to an impairment of proteasomal function. These data suggest that proteasomal dysfunction and ER-stress play an important role in the pathogenesis of LD.

## Results

### 1.- Lack of laforin enhances cell sensitivity to agents that induce endoplasmic reticulum stress

**Figure 1:**
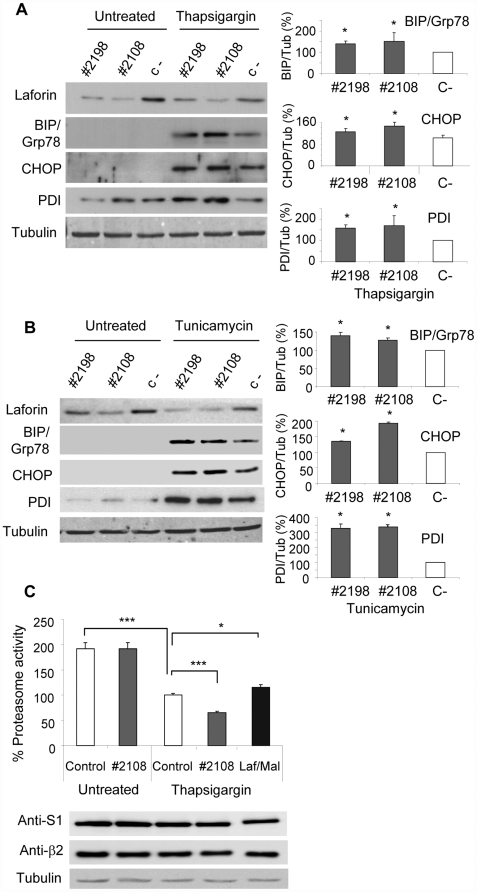
Lack of laforin enhances cell sensitivity to endoplasmic reticulum stress in Hek293 cells. A) Western-blot analysis of homogenates from Hek293 cells treated with different siRNA against laforin (#2198 and #2108) or control siRNA (C-). Thirty hours after silencing, cells were cultured either in presence or absence of 1 µM thapsigargin for 18 h. A representative blot of three independent experiments is shown. Right panel shows quantification (mean±SEM) of different blots expressed in percentage respect to cells treated with control siRNA (100%). Tubulin was used as a loading control. B) Similar analysis as in A) but in cells treated with tunicamycin (2 µg/ml, 18 h). C) Proteasome activity was assayed in Hek293 cells under different treatments as above by using a luminogenic proteasome substrate (succinyl-leucine-leucine-valine-tyrosine-aminoluciferine) and referred to the values obtained in control cells treated with thapsigargin. The effect of laforin was assayed by either depleting endogenous laforin (#2108) or by overexpressing laforin and malin (Laf/Mal). Cell extracts (50 µg) were also analyzed by western blotting using antibodies against two different subunits of the 26S proteasome (anti-S1 and anti-β2) (lower panel).

To test the possible involvement of laforin in the ER-unfolded protein response pathway (UPR), we depleted Hek293 cells of laforin by siRNA silencing. As shown in Fig. 1A (upper panel), siRNA oligos #2198 and #2108 (see Materials and Methods) depleted endogenous laforin in Hek293 cells, with oligos #2108 showing higher efficiency. We then checked for the presence of UPR-markers such as the heat shock protein BIP/Grp78 and the transcriptional factor CHOP (see Introduction). Under standard growth conditions, short-term laforin depletion did not increase the levels of these markers. However, if cells were treated with thapsigargin, an ER-stress producing agent ([26], [27], [28]), laforin-depleted cells accumulated significantly higher levels of BIP/Grp78 and CHOP than control non-depleted cells (see quantification of the levels of the proteins in the right panel of Fig. 1A). We also measured the levels of another ER-stress marker involved in oxidative stress, PDI (protein disulfide isomerase). The levels of this marker also increased in laforin-depleted cells treated with thapsigargin (Fig. 1A). These results indicated that laforin prevented the induction of ER-stress markers upon thapsigargin treatment. Similar results were obtained upon treatment of the cells with tunicamycin, another ER-stress producing agent (Fig. 1B).

As proteasome function plays a prominent role in the UPR pathway ([26], [27], [28]), we measured the proteasomal activity in cells depleted or not of laforin and subjected to conditions of ER-stress (treatment with thapsigargin). As shown in Fig. 1C, depletion of laforin did not affect the activity of the proteasome when the cells were growing under standard conditions. Upon thapsigargin treatment, control (non-depleted) cells showed a clear decrease in the activity of the proteasome, in agreement with previous reports ([26], [27], [28]). However, the activity of the proteasome was significantly lower in cells depleted of laforin (oligo #2108) (Fig. 1C). These results indicated that under conditions of ER-stress, loss of laforin impaired proteasomal function. Consistent with a protective role of laforin on the activity of the proteasome, overexpression of laforin and malin preserved to some extent the activity of the proteasome in control cells treated with thapsigargin (Fig. 1C). Western blot analyses of two different subunits of the 26S proteasome complex indicated that the observed changes in the activity of the proteasome were not due to differences in proteasome levels (Fig. 1C, lower panel).

**Figure 2:**
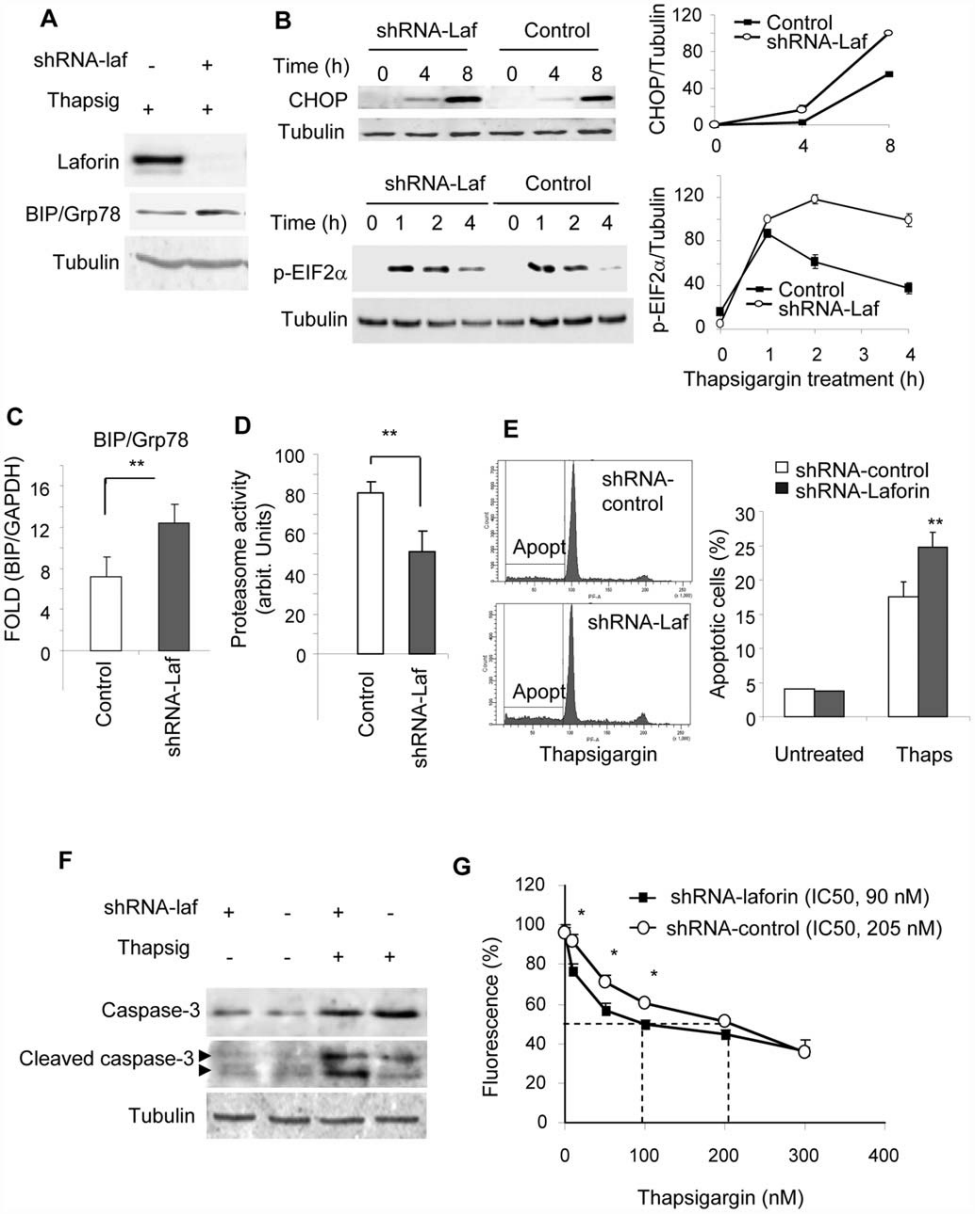
Lack of laforin enhances cell sensitivity to endoplasmic reticulum stress in human neuroblastoma SH-SY5Y cells. SH-SY5Y cells were stably transfected with empty pSuper.neoGFP vector or pSUPER-laforin expressing a shRNA against laforin, as described in Materials and Methods. A) Stable transfectants were treated with 1 µM thapsigargin for 18 h and analyzed by western-blot using anti-BIP/Grp78, anti-laforin and anti-tubulin antibodies. B) The expression of additional ER-stress markers such as CHOP and pEIF2α (phospho-EIF2alpha) was analyzed in the stable transfectants at different times after the treatment with 1 µM thapsigargin (see Supplementary Fig. S1 for time course appearance of different ER-stress markers); right panel shows quantification (mean±SEM) of different blots expressed as percentage respect to tubulin lebels. C) Quantitative real time PCR analysis of the expression of BIP/Grp78 in stable transfectants treated with 1 µM thapsigargin for 18 h; expression of target gene was normalized using GAPDH as an internal control; data are expressed as fold induction over untreated control (mean±SEM) of four independent measurements. D) Proteasome activity was measured in extracts from transfectants used in A) treated with 1 µM thapsigargin. E) Growth of control and laforin depleted transfectants was assessed by flow cytometry as described in Materials and Methods. The percentage of apoptotic cells in the sub-G_1_ population was measured in cells treated or not with 1 µM thapsigargin for 18 h. The left panel shows a representative analysis of three independent experiments of cells treated with thapsigargin; the right panel shows the corresponding mean±SEM. F) Extracts from laforin-depleted and control cells treated or not with 1 µM thapsigargin for 18 h were analyzed by western-blotting using anti-caspase 3 and anti-activated caspase 3 antibodies; tubulin was used as a loading control. G) Cell sensitivity of laforin depleted and non-depleted cells to thapsigargin treatment. Stable laforin depleted SH-SY5Y and control cells were cultured in 96-well plates and treated with different amounts of thapsigargin for 24 hr. Then, cell viability was assessed using the AlamarBlue assay as described in Materials and Methods. Each point represents mean±SEM of three independent measurements and expresses the percentage of viability respect to the corresponding untreated cells.

Because LD is a neurological disorder, we sought to replicate the experiments in a human neuroblastoma cell line (SH-SY5Y). In addition, we decided to study the effect of long-term laforin depletion. To this end, we constructed a plasmid expressing a shRNA based on oligo #2108 (pSUPER-Laf) that was introduced in SH-SY5Y cells to select stable transfectants. These stable transfectants were grown in selective media for five days and, as shown in Fig. 2A, they presented undetectable levels of laforin. We then measured the amount of BIP/Grp78 under untreated and ER-stress conditions (induced by thapsigargin). As shown in Fig. 2A, we found a significant increase in the levels of this marker in comparison to stable transfectants obtained with an empty plasmid, but only when the cells were under conditions of ER-stress (thapsigargin treatment). The higher levels of expression of BIP/Grp78 in laforin-depleted cells treated with thapsigargin were confirmed by quantitative real time PCR (Fig. 2C). In addition, we measured the levels of additional ER-stress markers such as CHOP and phospho-EIF2alpha. As shown in Fig. 2B, higher levels of these two markers were found in laforin-depleted cells treated with thapsigargin. We also measured the activity of the proteasome in these cells and found a significant decrease in its activity in SH-SY5Y laforin-depleted cells subjected to conditions of ER-stress (thapsigargin treatment) (Fig. 2D). Similar results were obtained in two independent stable transfectants; not shown.

As it has been described that continued conditions of ER-stress lead to apoptotic cell death ([26], [27], [28]), we used flow cytometry to determine the percentage of apoptotic cells in SH-SY5Y cultures of laforin-depleted and control non-depleted cells. As shown in Fig. 2E, there was a significant increase in the percentage of apoptotic cells in the cultures of laforin-depleted cells in comparison to control cells but only when the cultures were subjected to conditions of ER-stress. In agreement with these results, we observed an increase in the levels of endogenous activated-caspase 3 in laforin-depleted cells treated with thapsigargin (Fig. 2F). In addition, we determined the sensitivity of laforin-depleted and control cells to thapsigargin treatment. As it can be observed in Fig. 2G, laforin-depleted cells were more sensitive to thapsigargin (IC50, 90 nM) in comparison to control cells (IC50, 205 nM).

As a whole, these results indicate that cells lacking laforin are more sensitive to conditions of ER-stress and present an impairment of the ubiquitin-proteasomal function. This combination of effects is likely to originate a more severe ER-stress response which may culminate in increased apoptosis.

### 2.- Analysis of ER-stress markers in LD patients and in laforin knockout mice (Epm2a-/-)

**Figure 3:**
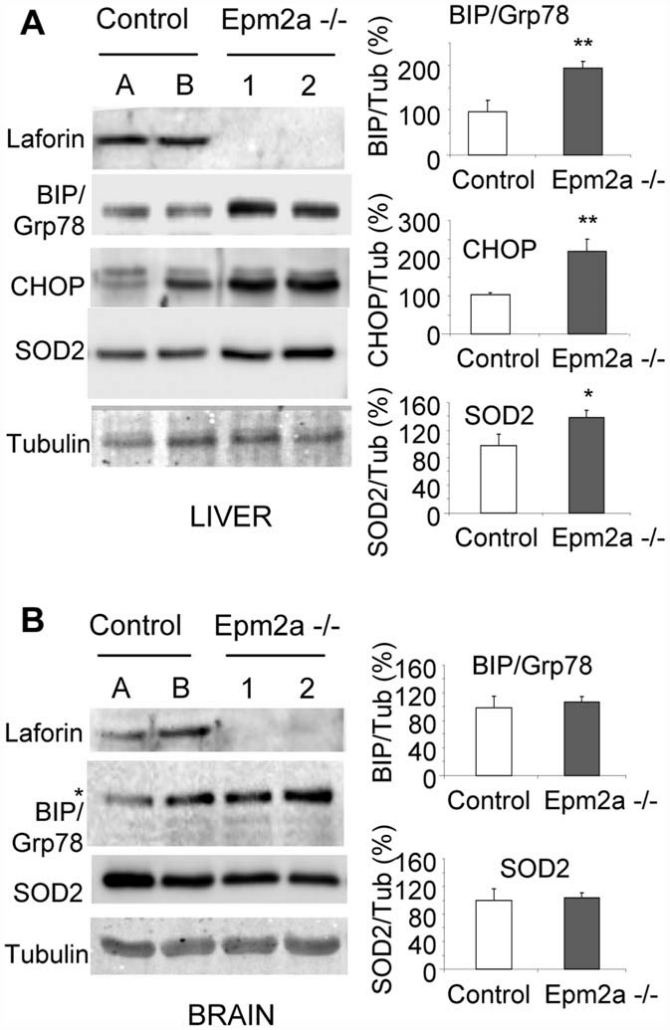
Laforin deletion results in increased ER stress in the liver of Epm2a-/- mice. A and B) Western blot analyses of laforin and ER-stress markers (BIP/Grp78, CHOP, SOD2) in liver (A) and whole brain (B) biopsies of two Epm2a+/+ (A and B) and two Epm2a-/- mice (1 and 2). A representative blot of four different animals of each type is shown. Right panels show normalized intensities (mean±SEM; n: 4) of different markers expressed as a percentage with respect to control mice. Tubulin was used as a loading control. * In brain samples, a band of 94 kDa is recognized by the anti-BIP/Grp78 antibody instead of the corresponding 78 kDa band.

**Figure 4:**
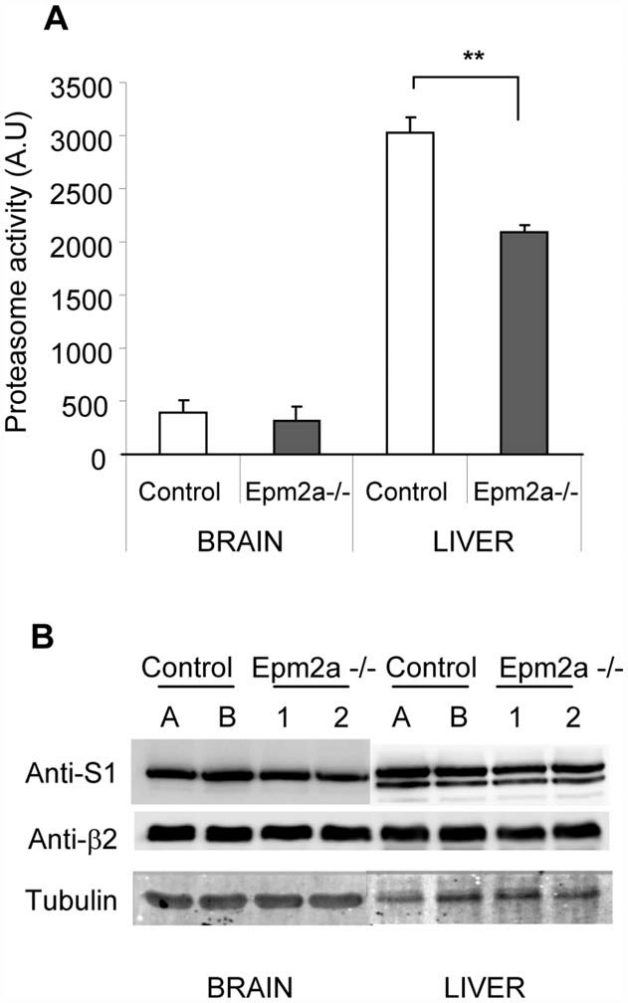
Proteasome activity in biopsies of Epm2a+/+ and Epm2a-/- mice. A) Proteasome activity was assessed by using the luminogenic proteasome substrate (succinyl-leucine-leucine-valine-tyrosine-aminoluciferine) in extracts from whole brain and liver of Epm2a+/+ and Epm2a-/- mice as described in Materials and Methods. Data are presented as luciferase activity (mean±SEM; n: 4), and represent the average of two luciferase activity determinations for each sample. B) Fifty micrograms of total lysates from liver and whole brain biopsies of two Epm2a+/+ (A and B) and two Epm2a-/- mice (1 and 2) were analyzed by SDS–PAGE and western blotting using anti-S1 (19S regulatory particle), anti-β2 (20S proteasome) and anti-tubulin antibodies.

To confirm the results obtained with the cell line models (see above) in laforin knockout (Epm2a-/-) mice, several markers of ER-stress were analyzed in extracts from different tissues of 9-month old mice. As expected RT-PCR analysis indicated that the Epm2a-/- mice, kindly provided by Dr. Delgado-Escueta, lacked laforin expression because of the described deletion of the *EPM2A* gene [21] (not shown), and antibodies against laforin were unable to detect any band in crude extracts from different tissues of these animals (Fig. 3). In agreement with data obtained with Hek293 and SH-SY5Y cells, extracts from the liver of Epm2a-/- mice contained higher levels of BIP/Grp78 and CHOP compared to age and sex matched C57BL6 control mice (Fig. 3A). In addition, we also detected higher levels of SOD2 (superoxide dismutase 2), another ER-stress marker related to oxidative stress (Fig. 3A). These data indicated that the liver of Epm2a-/- animals, lacking laforin, was under permanent conditions of ER-stress. In contrast, analysis of whole brain extracts from these animals failed to detect differences in the levels of the ER-stress markers between Epm2a-/- and control animals (Fig. 3B). We also measured the activity of the proteasome in mouse liver and whole brain extracts. As shown in Fig. 4A, we observed a significant decrease in the activity of the proteasome in liver extracts from Epm2a-/- mice in comparison to liver extracts from control mice. However, we were not able to observe differences in the activity of the proteasome in whole brain extracts, which correlated with the absence of differences in the amount of ER-stress markers in extracts from this tissue (see above). Western blot analyses of two different subunits of the 26S proteasome complex in these liver samples indicated that the observed changes in the activity of the proteasome were not due to differences in proteasome levels (Fig. 4B). These results supported the idea that loss of laforin correlated with induction of ER-stress markers and impaired proteasomal function, at least in mouse liver.

**Figure 5:**
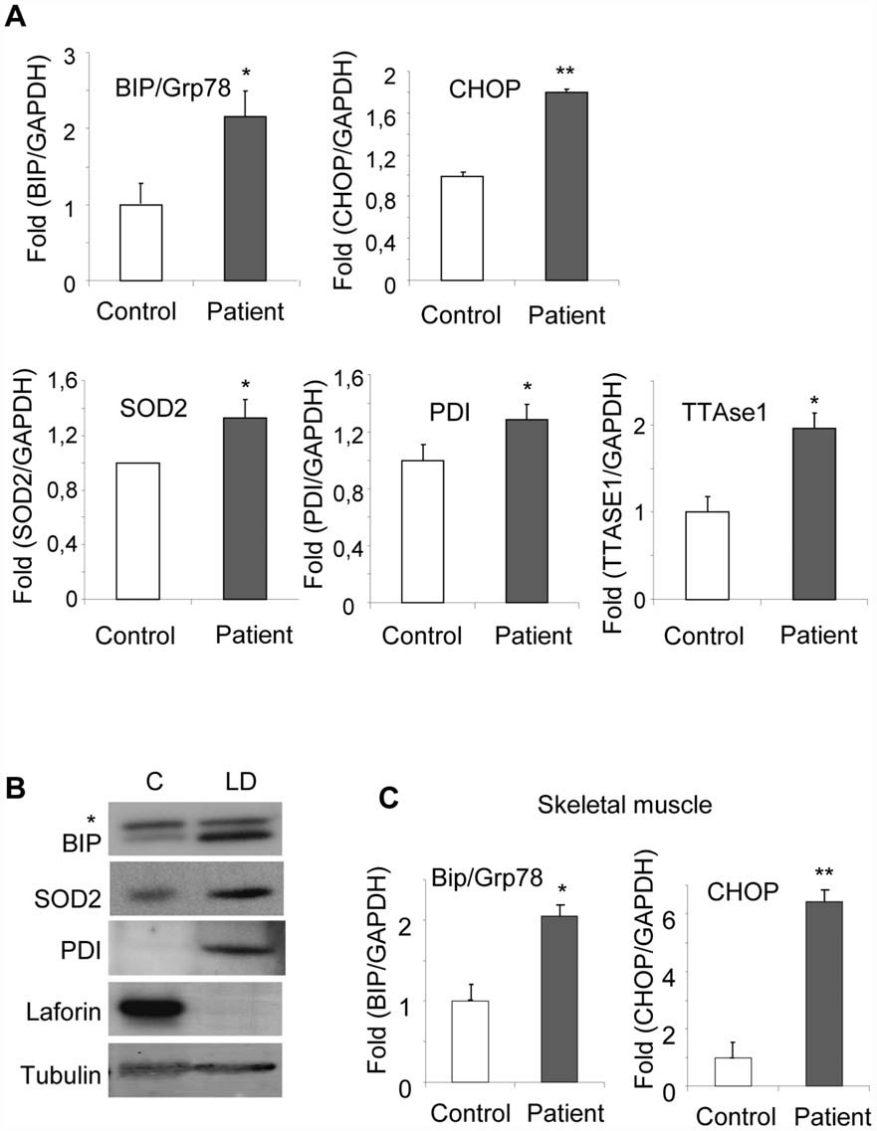
Expression of ER-stress markers is increased in brain necropsies of a LD patient. A) Quantitative-real time PCR analysis of ER-stress markers in brain necropsies from both control and LD patient. Expression of target genes was normalized using GAPDH as an internal control. Data are expressed as fold induction over control (mean±SEM) of four independent measurements. B) Extracts from brain necropsies from both control and LD patient were analyzed by western blot using the indicated antibodies. Membranes were probed with anti-tubulin antibodies to ensure equal protein loading. * non-specific band. C) Quantitative-real time PCR analysis of ER-stress markers in skeletal muscle necropsies from both control and LD patient. Expression of target genes was normalized using GAPDH as an internal control. Data are expressed as fold induction over control (mean±SEM) of four independent measurements.

We also analyzed the levels of different ER-stress markers in a brain necropsy from an LD patient with mutations in the *EPM2A* gene (compound heterozygous with R241Stop and ex1-33bpdel mutations). Using quantitative real time PCR, we detected higher levels of mRNAs corresponding to BIP/Grp78 and CHOP, in comparison to samples obtained from necropsies of an age-matched control subject (Fig. 5A). In addition, we also detected higher levels of mRNAs corresponding to SOD2 (superoxide dismutase 2), PDI (protein disulphide isomerase) and TTAse1 (thioltransferase 1), three ER-markers related to oxidative stress (Fig. 5A). In agreement with these results, western blot analysis confirmed the presence of higher levels of BIP/Grp78, PDI and SOD2 in brain tissue samples from the LD patient (Fig. 5B). The expression of ER-stress markers (i.e., BIP/Grp78 and CHOP) was also increased in skeletal muscle samples from the same patient in comparison to a control subject (Fig. 5C).

All these *in vivo* data illustrated that in cells lacking laforin there was an induction of ER-stress markers and impaired proteasomal function that maintained these cells are under permanent conditions of ER-stress.

### 3.- Malin is overexpressed under conditions of ER-stress

**Figure 6:**
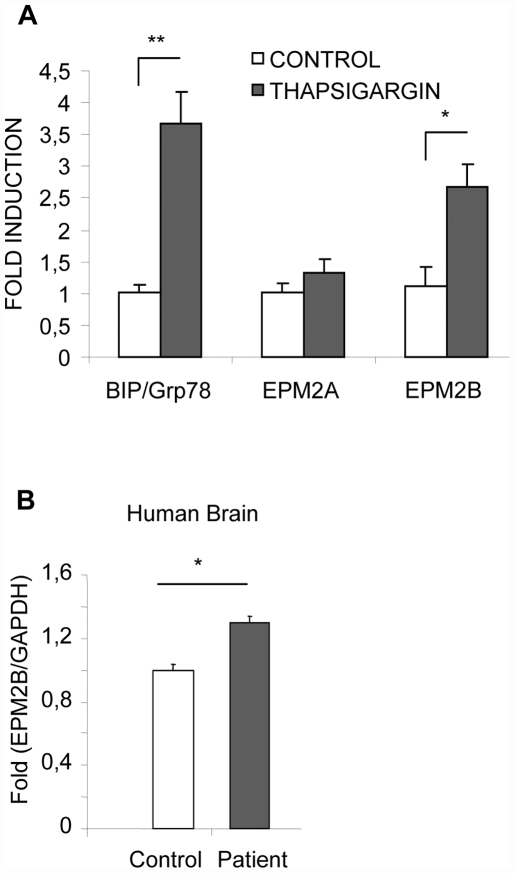
Malin expression is induced under conditions of ER-stress. A) SH-SY5Y cells were treated or not with 1 µM thapsigargin for 18 h and levels of mRNAs were measured by quantitative-real time PCR. Expression of target genes was normalized using GAPDH as an internal control. Data are expressed as fold induction over untreated cells (mean±SEM) of three independent measurements. B) Quantitative-real time PCR analysis of *EPM2B* gene expression in brain necropsies from both control and LD patient normalized using GAPDH as an internal control. Data are expressed as fold induction over control (mean±SEM) of two independent measurements.

As described above, the expression of ER-stress markers is induced under conditions of ER stress. In agreement with this, expression of the ER-stress marker BIP/Grp78 was increased in human neuroblastoma SH-SY5Y cells under conditions of ER-stress (Fig. 6A). We checked then whether the expression of laforin was also affected under ER-stress conditions but we did not observe any change in the expression of the *EPM2A* gene in these cells, treated or not with thapsigargin (Fig. 6A). This result was consistent with the absence of specific ER-stress responsive sites at the promoter region of the *EPM2A* gene (not shown). However, when we analyzed the expression of the gene encoding malin (*EPM2B*), the E3-ubiquitin ligase that interacts with laforin to form a functional complex (see Introduction), we found a significant increase in the expression of this gene in SH-SY5Y cells treated with thapsigargin (Fig. 6A). This observation was consistent with the presence of a putative ATF6 site (position -100/-79 respect to the ATG) in the promoter of the *EPM2B* gene (determined by using the Mat Inspector 7.4 software). Since, as we have described above, tissues from human and mouse lacking laforin were under conditions of permanent ER-stress, we therefore, measured the expression of the *EPM2B* gene by quantitative real time PCR in the brain tissue sample of the LD patient and observed a significant increase in the expression of the *EPM2B* gene compared to tissues from normal individuals (Fig. 6B). Based on these results, we suggest that malin could be considered as a novel marker of ER dysfunction.

## Discussion

Endoplasmic reticulum (ER) is the site where membrane and secretory proteins are folded and processed (addition of carbohydrate moieties, cis-trans isomerization of peptide bonds, arrangement of disulphide bonds, etc.). ER function is highly sensitive to stresses that perturb cellular energy levels, the red-ox state or Ca++ concentration. Such stresses reduce the folding capacity of the ER, which results in the accumulation and aggregation of unfolded/misfolded proteins. These unfolded proteins accumulate in the lumen of the ER triggering a signal responsible for the activation of the unfolded protein response (UPR). In addition, unfolded proteins are retrotranslocated to the cytosol, polyubiquitinated and degraded by the proteasome by the ER-associated degradation (ERAD) pathway. UPR and ERAD are highly coordinated processes: efficient ERAD requires intact UPR and UPR induction increases ERAD capacity. However, when UPR and ERAD are impaired, cell viability is markedly decreased ([31], [32]). The last steps of the ERAD pathway involve ubiquitination and proteasomal degradation of unfolded substrates. In mammals, several integral ER-membrane proteins with E3-ubiquitin ligase activity, such as synoviolin/HsHrd1 and gp78, play a role in ERAD. In addition, several soluble cytosolic E3-ubiquitin ligases, such as parkin and CHIP (carboxy terminus of the Hsc70-interacting protein), also function in ERAD. All these E3-ubiquitin ligases ubiquitinate unfolded proteins marking them for proteasomal degradation. Defects in the function of any of these E3-ubiquitin ligases results in proteasomal dysfunction and in the accumulation of specific unfolded proteins, which triggers the UPR ([33], [34], [35], [36]). Similarly, in this work we present evidence indicating that in the absence of laforin, cells become more sensitive to agents triggering ER-stress and that this situation correlates with an impairment of proteasomal function. Conversely, the overexpression of laforin resulted in a partial prevention of proteasomal dysfunction upon ER-stress conditions. It is worth pointing out that proteasomal activity is not affected by depletion of laforin in the two cell lines used in this study, unless cells were subjected to ER-stress. This could indicate that only when cells are challenged with ER-stress conditions, the presence of laforin exerts its protective role, what suggests that the function of laforin, in combination with its partner malin (E3-ubiquitin ligase), would be to contribute to the elimination of unfolded proteins upon ER-stress conditions. In agreement with this hypothesis, it has been very recently described that the laforin-malin complex suppresses the cellular toxicity of misfolded proteins by promoting their degradation through the ubiquitin-proteasome system [37]. Therefore, we suggest that in the absence of a functional laforin/malin complex more unfolded proteins would accumulate causing proteasomal dysfunction and aggravating the conditions of ER-stress. However, liver extracts of Epm2a-/- mice already presented lower proteasomal activity and higher induction of ER-stress markers than control mice. We suggest that during the life of the mice used in this study (9 months), they have been probably subjected to environmental situations that may have elicited conditions of ER-stress. The absence of laforin in these animals would have prevented them from recovering from these challenges, resulting in the establishment of permanent ER-stress conditions.

As indicated above, liver cells of Epm2a-/- mice present constitutive conditions of ER-stress. By analogy, human LD patients lacking laforin might also present permanent ER-stress conditions in the liver and other tissues, which may explain some cases of LD patients in whom liver failure has anticipated the neurological symptoms [38]. However, brain extracts from Epm2a-/- mice do not present any detectable alteration in the regulation of the UPR and proteasomal function. Since we present evidence that in the brain of a human LD patient there is a clear induction of ER-stress markers, we suggest that differences between samples from mouse and human brains may account for this apparent contradiction. For example, mice and human may have a distinct progression of the disease [21], (i.e., the disease could not have been established in the brain of the mice, yet), or these tissues may have been differentially exposed to ER-stress conditions.

It is known that prolonged conditions of ER-stress triggers apoptosis, as a last resort of multicellular organisms to dispense of dysfunctional cells. Since we present evidence that samples from the brain of a human LD patient lacking laforin presented increased levels of ER-stress markers, this suggests that LD cells may also be more sensitive to apoptosis. In agreement with this hypothesis we found higher levels of apoptosis in a human neuroblastoma cell line (SH-SY5Y) depleted of laforin when subjected to ER-stress conditions. Moreover, it was recently described that 2-deoxyglucose treatment (causing ER-stress because of energy deprivation) also induces higher levels of apoptosis in cells defective of laforin than in normal cells [39]. This enhanced predisposition to apoptosis could be deleterious for the central nervous system and could lead to increased neurodegeneration.

As described above, the laforin/malin complex has a protective role in ER-stress. This protective role likely overlaps the role of other cytosolic E3-ubiquitin ligases involved in ERAD, such as parkin and CHIP. Defects in the function of parkin and CHIP results in an impairment of proteasomal function and overexpression of these proteins results in an amelioration of ER-stress conditions ([40], [41], [42]). These are situations that closely resemble the laforin data reported here. Moreover, malin expression, similarly to parkin and CHIP, is increased under conditions of ER-stress. All these results suggest that the laforin/malin complex may be a novel component of the ERAD pathway. In this sense, the recent report that indicates that the laforin-malin complex is involved in the degradation of misfolded proteins [37], would support this hypothesis. Since parkin and CHIP target a specific set of unfolded proteins for degradation, further studies would be needed to determine whether this is also the case for the laforin-malin complex and to characterize its physiological substrate(s). In any case, our results indicate that Lafora disease may be considered as a novel pathology related to ER-stress, which may offer novel opportunities for therapeutic developments.

## Materials and Methods

### Ethics statement

All animal care and use procedures used in this study were in accordance with the guidelines of the Institutional Animal Care and Use Committee and approved by the Centro de Investigaciones Biológicas (CSIC) ethical review board. Tissues from post-mortem examination of the LD patient were obtained with explicit written informed consent for their preservation and future use in studies like those reported in this work. Written consent was also given by the control individual to collection and use of tissues in research. The use of these human tissues in this study was approved by the Centro de Investigaciones Biologicas (CSIC) ethical review board.

### Cell culture and treatments

Hek293 or SH-SY5Y cells were grown in DMEM containing 25 mmol/l glucose, supplemented with 10% fetal bovine serum (FBS) or in DMEM:F12 with 15%FBS respectively, with antibiotics (100 U/ml penicillin, and 100 µg/ml streptomycin) at 37°C in an atmosphere of humidified 5% CO_2_. When indicated cells were treated with 1 µM thapsigargin or 2 µg/ml tunicamycin. After treatment, cells were harvested and total cell extracts were subjected to western blot analysis or proteasome activity quantification as indicated.

### siRNA laforin silencing

Two different siRNA oligonucleotides were designed for laforin (#2198-sense, 5′- GGUAAUAAUUGGUAUUCAGtt-3′ and #2198-antisense 5′- CUGAAUACCAAUUAUUACCtc-3′) and (#2108-sense, 5′-GGUGGAACAUGUAACCAUCtt-3′ and #2108-antisense, 5′- GAUGGUUACAUGUUCCACCtg-3′) (Ambion. Austin, TX, USA). Cells were treated in parallel with a control siRNA (Ambion. Austin, TX, USA). Cells were transfected in at a confluence of 70%. Specific laforin siRNA or negative control siRNA (100 nM) were mixed with Lipofectamine 2000 (Invitrogen, Carlsbad, CA. USA) according to manufacturer's recommendation and added to the cells. After 3 h 30 min at 37°C, the medium was changed, and the cells were cultivated as indicated above.

The mammalian expression vector, pSUPER.neo.GFP (Oligoengine, Seattle, USA) was used for expression of shRNA targeting laforin in human neuroblastoma SH-SY5Y cells. Forward and reverse synthetic 64 nt oligonucleotides containing sense (19 nt), nt stem-loop and anti-sense (19 nt) nucleotides corresponding to nucleotides 872–891 of human EPM2A cDNA (GenBank accession no. NM_005670), were annealed and subcloned into pSUPER.neo.GFP vector yielding plasmid pSUPER-laforin. Empty plasmid and pSUPER-laforin were linearized by digestion with *Sca*I and used to transfect cells with Lipofectamine 2000 (Invitrogen). Selection of drug-resistant colonies was performed by addition of G418 (600 µg/ml active concentration) 48 h after transfection; the same amount of the antibiotic was maintained throughout culture. Resistant clones were screened by flow cytometry following GFP expression. Western blot analysis showed that pSUPER.neo.GFP transfectants had normal laforin levels whereas pSUPER-laforin transfectants showed substantial laforin depletion.

### Apoptosis and cell viability assays

Apoptosis was determined by flow cytometry as the amount of cells in the sub-G1 peak after propidium iodide staining of ethanol fixed cells. Briefly, cells were harvested by centrifugation at 2000 ×*g* for 5 min. The pellet was washed once with PBS, resuspended in 70% EtOH and kept overnight at 4°C. Fixed cells were pelleted and stained for 30 min at 37°C with 20 µg/ml propidium iodide in PBS containing 10 µg/ml RNase A. Data were collected with the FACSCanto system (Becton Dickinson) and analyzed with FACS DIVA software. Additionally, protein extracts were analyzed by western-blotting using anti-caspase 3 and anti-activated caspase 3 antibodies (Cell Signaling Technology, MA).

Cell viability was determined in SH-SY5Y cells stably transfected with either pSUPER.neo.GFP or pSUPER-laforin. 1.5×10^4^ cells were seeded in 96-well plastic plates the day before treatment. Cells were treated with thapsigargin at concentration ranging from 0 to 300 nM and 24 h later, cell viability was evaluated by using the AlamarBlue oxidation reduction indicator (Serotec Ltd., Oxford, United Kingdom) following manufacture's instructions. Fluorescence measurements were taken at 600 nm with a Fuji FLA5000 system. The viability of each culture was calculated as a percentage respect to an untreated control.

### Laforin deficient mice (Epm2a-/-)

Laforin deficient Epm2a-/- mice were kindly provided by Dr Antonio V. Delgado-Escueta [21]. Male C57BL6 wild type and isogenic Epm2a-/- mice were maintained at the Centro de Investigaciones Biológicas (CSIC, Madrid) on a LD12:12 cycle under constant temperature (23°C) with free access to food and water. Genotypes were determined as described in [21]. Nine-month-old knockout or control mice of the same age were sacrificed by cervical dislocation, liver and brain tissues were dissected and tissue extracts were obtained as described below.

### Case report and necropsies

Necropsies of different human tissues, including brain, were obtained from a laforin-deficient LD patient and a control individual 24 hours after death. The specimens were stored in 50 ml tubes in phosphate-buffered saline at 4°C during transportation. Tissue samples were divided into small pieces and frozen at −80°C immediately after arrival at the laboratory. The LD patient was previously characterized as a compound heterozygote for the *EMP2A* mutations R241Stop and ex1-33bpdel.

### Obtaining cell extracts and immunodetection

Tissue lysates (from mice and human samples) were prepared by homogenizing samples with a tissue homogenizer in ice-cold lysis buffer containing 0.15 M NaCl, 10 mM Tris-HCl pH 7.5, 15 mM EDTA pH 8.0, 0.6 M sucrose, 0.5% NP-40, 50 mM NaF, 5 mM Na_2_P_2_O_7_, 1 mM PMSF and protease inhibitor cocktail (Roche). Cell line lysates were obtained by repeated passage through a 25-gauge needle in ice-cold lysis buffer. Protein content was determined using the Bradford method (Bio-Rad). Fifty µg of total protein from the soluble fraction of lysates were analyzed by SDS-PAGE and western blotting using appropriate antibodies. Blocking was performed in 5% nonfat milk for 1 h at room temperature, except for anti-CHOP, anti-S1 and anti-β2 in which 5% BSA in PBS-T (phosphate buffer saline plus 0.1% Tween 20) was used. Blots were probed with indicated antibodies at 1∶1000 dilution in TBS-T (50 mM Tris-HCl, 154 mM NaCl, pH 7.5 plus 0.1% Tween 20) plus 5% nonfat milk. Polyclonal antibodies against PDI, BIP/Grp78, phospho-EIF2alpha (Ser51) (Cell Signaling Technology, MA); GADD153/CHOP, SOD2 (Santa Cruz Biotechnology, Inc., Santa Cruz, CA, U.S.A.); anti-S1 (19S regulatory particle) and anti-β2 (20S proteasome) (Biomol Research Labs; Exeter, UK) were used in the analyses. Mouse anti-laforin (monoclonal antibody against human laforin) was described previously [11]. Protein loading was assessed by reprobing the membrane with an anti-tubulin antibody (Santa Cruz Biotechnology, Inc) and IRDye 800/700–labelled secondary antibodies (1∶10000) (LI-COR Biosciences, Lincoln, NE). Visualization of protein expression was performed using the LI-COR Odyssey IR Imaging System.

### Proteasome activity quantification

40 µg of soluble protein extracts were incubated with the Promega Proteasome-Glo Assay Reagent (Promega Bioscience, Madison, WI) for 10 minutes. The chymotrypsin-like proteasome activity was detected as the relative light unit (RLU) generated from the cleaved substrate in the reagent. Luminescence generated from each reaction condition was detected with a Wallac 1420 VICTOR luminometer.

### Quantitative real-time PCR

Dissected brain biopsies were frozen in liquid nitrogen and stored at –80°C. Thawed tissue was homogenized in 2 ml TRIPURE reagent (Roche Diagnostics, Manheim, Germany) and total RNA was isolated according to the manufacturer's instructions. The integrity of total RNA was verified by electrophoresis through denaturing agarose gels. First strand cDNA was synthesized from 1 µg of total RNA using random hexamer and expand reverse transcriptase (Roche Diagnostics, Manheim, Germany). cDNA was used as a template for real-time PCR. PCR primers and fluorogenic TaqMan probe sets for each gene were designed using Universal probe library Service (Roche Molecular Biochemicals) to meet all TaqMan design guidelines. Probes were synthesized with a reporter dye 6-carboxyfluorescein (6-FAM) covalently linked at the 5′ end and a quencher dye 6-carboxy-tetramethyl-rhodamine (TAMRA) was linked to the 3′ end of the probe. See Table 1 for primer and probe sequences. Each PCR was carried out in a final volume of 25 µl of PCR Master Mix (Applied Biosystems), containing 200 nM of each primer and 40 nM of specific fluorescent probe. The cycle conditions were 20 s at 95°C for initial denaturing, followed by 35 cycles of 95°C for 3 s and 60°C for 30 s in the 7500 Fast Real-time PCR system (Applied biosystems). GAPDH was used as an internal standard. Each reaction was done in duplicate from at least three independent experiments. The relative amount of each mRNA was calculated using the second derivative comparative Ct method.

**Table 1:** Sequences of the primers and probes used in this work.

GENE	Primer FWD	Primer REV	TaqMan probe number
LAFORIN/EPM2A	cgtggacacgttctggtaca	cggtcatgatgaggtccatt	#52
GAPDH	ctctgctcctcctgttcgac	acgaccaaatccgttgactc	#60
CHOP	agctggaacctgaggagaga	tggatcagtctggaaaagca	#9
BIP/Grp78	agcctggcgacaagagtg	tccttgggcagtattggatt	#39
MALIN/EPM2B	gggctgagcctctactttcc	cctggtgatcaaaggtcaca	#16
PDI	gagctcctagcgggcttt	caccaccacgaacagcac	#16
SOD2	ctggacaaacctcagcccta	tgatggcttccagcaactc	#22
TTase1	ttggagctctgcagtaacca	atccaccagaagtgctgtca	#42

### Statistical analyses

Values are given as means±SEM of three independent experiments. Differences between groups were analyzed by two-tailed student's t-tests. The significance has been considered at * p<0.05, ** p<0.01 and *** p<0.001, as indicated in each case.

## Supporting Information

Figure S1 (8.28 MB TIF)
