# Supplementary figures and images for: Increased Endoplasmic Reticulum Stress and Decreased Proteasomal Function in Lafora Disease Models Lacking the Phosphatase Laforin
Source: PLoS One. 2009 Jun 16;4(6):e5907. doi: 10.1371/journal.pone.0005907 (PMC2692001; doi:10.1371/journal.pone.0005907)

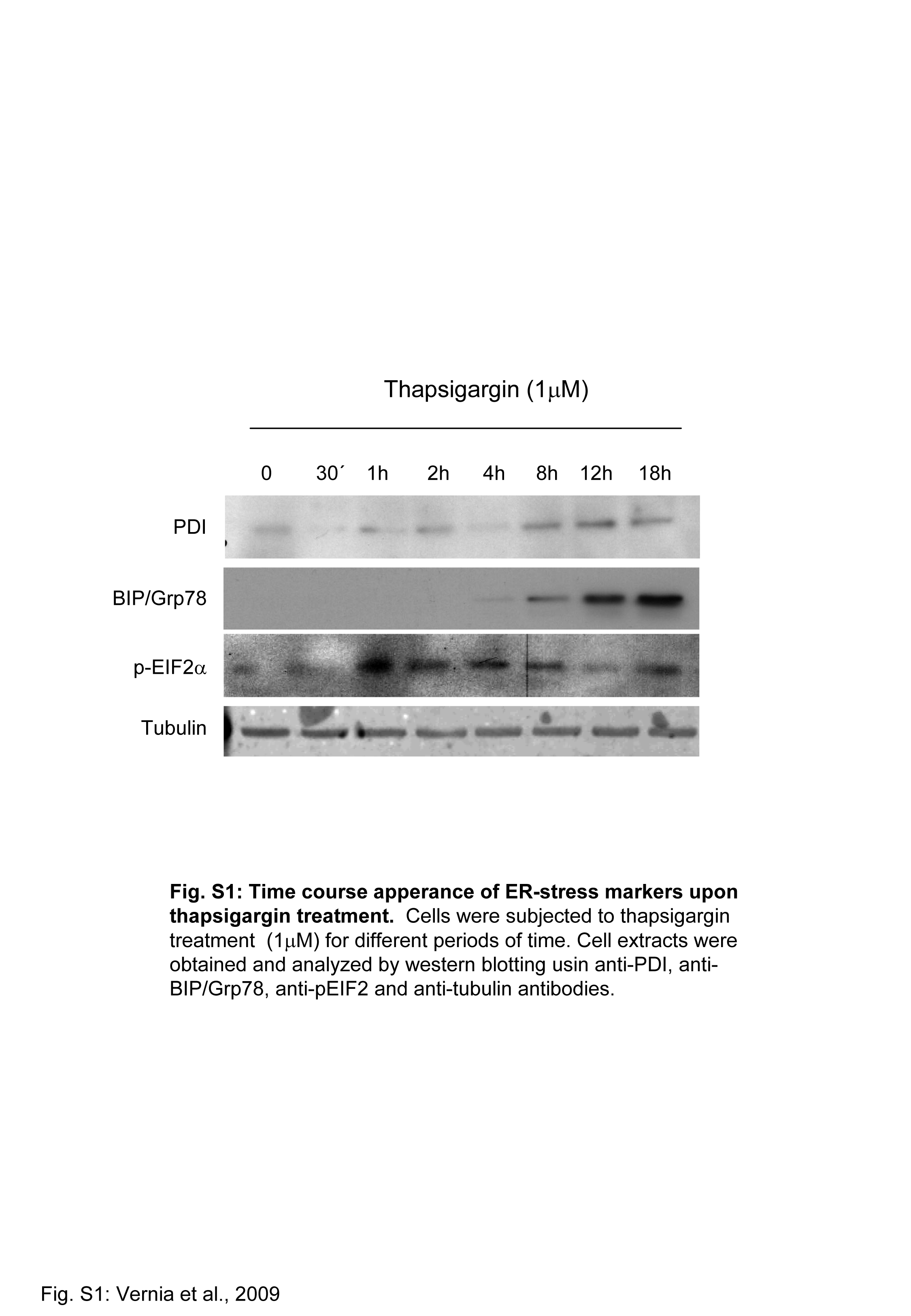

Supplement: Figure S1 — (8.28 MB TIF) [file pone.0005907.s001.tif]
